# Geminivirus-Mediated Genome Editing in Potato (*Solanum tuberosum* L.) Using Sequence-Specific Nucleases

**DOI:** 10.3389/fpls.2016.01045

**Published:** 2016-07-21

**Authors:** Nathaniel M. Butler, Nicholas J. Baltes, Daniel F. Voytas, David S. Douches

**Affiliations:** ^1^Department of Plant, Soils and Microbial Sciences, Michigan State University, East LansingMI, USA; ^2^Department of Genetics, Cell Biology and Development and Center for Genome Engineering, University of Minnesota, MinneapolisMN, USA

**Keywords:** bean yellow dwarf virus, gene targeting, homologous recombination, gene replacement, acetolactate synthase, CRISPR/Cas, TALEN, ZFN

## Abstract

Genome editing using sequence-specific nucleases (SSNs) is rapidly being developed for genetic engineering in crop species. The utilization of zinc finger nucleases (ZFNs), transcription activator-like effector nucleases (TALENs), and clustered regularly interspaced short palindromic repeats/CRISPR-associated systems (CRISPR/Cas) for inducing double-strand breaks facilitates targeting of virtually any sequence for modification. Targeted mutagenesis via non-homologous end-joining (NHEJ) has been demonstrated extensively as being the preferred DNA repair pathway in plants. However, gene targeting via homologous recombination (HR) remains more elusive but could be a powerful tool for directed DNA repair. To overcome barriers associated with gene targeting, a geminivirus replicon (GVR) was used to deliver SSNs targeting the potato *ACETOLACTATE SYNTHASE1* (*ALS1*) gene and repair templates designed to incorporate herbicide-inhibiting point mutations within the *ALS1* locus. Transformed events modified with GVRs held point mutations that were capable of supporting a reduced herbicide susceptibility phenotype, while events transformed with conventional T-DNAs held no detectable mutations and were similar to wild-type. Regeneration of transformed events improved detection of point mutations that supported a stronger reduced herbicide susceptibility phenotype. These results demonstrate the use of geminiviruses for delivering genome editing reagents in plant species, and a novel approach to gene targeting in a vegetatively propagated species.

## Introduction

Genome editing is rapidly becoming a standard tool for genetic improvement in crop species. Major advancements in sequence-specific nuclease (SSN) technology, such as zinc finger nucleases (ZFNs), transcription activator-like effector nucleases (TALENs), and clustered regularly interspaced short palindromic repeats (CRISPR)/CRISPR-associated systems (Cas) has enabled application of genome editing in a range of crops species and development of transgene-free genetically engineered crops (Lusser et al., 2012; Palmgren et al., 2014). Genome editing allows generation of transgene-free genetically modified events by utilizing DNA repair pathways and modifying target DNA in *trans* without relying on stable integration of genome editing reagents (Curtin et al., 2012).

One method of genome editing involves the induction of DNA double-strand breaks by expressing SSNs *in vivo*. Once double-strand breaks have formed, DNA repair follows two major pathways: non-homologous end-joining (NHEJ) and homologous recombination (HR). NHEJ repair is often imprecise and can result in the introduction of insertions or deletions at the break site which can be used for targeted mutagenesis of endogenous genes or reporters (Li et al., 2012; Zhang et al., 2013). HR is a more precise pathway that utilizes a DNA template for repair. The ability to manipulate an exogenously supplied homologous repair template allows incorporation of new sequence at or near a break site in a process referred to hereafter as gene targeting (Puchta et al., 1996; Bibikova et al., 2003). Gene targeting provides many advantages over targeted mutagenesis but occurs at low frequencies in plant cells (Lee et al., 1990; Terada et al., 2002; Shukla et al., 2009).

Plant viruses have the potential to become powerful tools for genome editing in plants. For decades, mammalian viruses have been used for gene therapy in humans by directing high expression of genome editing reagents in pathological tissues (Giacca and Zacchigna, 2012; Kotterman and Schaffer, 2015). More recently, plant viruses such as the *Tobacco rattle virus* (*TRV*) have been used with *Agrobacterium tumefaciens* to efficiently deliver RNA interference (RNAi) reagents, ZFNs, and single-guide RNA (sgRNA) used in CRISPR/Cas for genetic modification in Solanaceous species, petunia (*Petunia hybrid*) and tobacco (*Nicotiana tabacum*; Brigneti et al., 2004; Burch-Smith et al., 2006; Marton et al., 2010; Sha et al., 2014; Ali et al., 2015). The limitations in carrying capacity of *TRV* and other RNA viruses prevent their use beyond expression of relatively small SSNs and sgRNAs and are unable to efficiently deliver DNA repair templates.

Geminiviruses may be able to overcome the limitations of RNA viruses by allowing a larger carrying capacity and producing a DNA replicon capable of acting as a repair template for gene targeting (Baltes et al., 2014; Richter et al., 2014). As a DNA virus, geminiviruses such as the bean yellow dwarf virus (BeYDV) replicate within the plant nucleus through a double-stranded intermediate to a high-copy number using host polymerases (Liu et al., 1997, 1998). The *BeYDV* genome encodes relatively few *cis*-acting elements and requires only one geminivirus *trans*-acting element for efficient replication (Rep/RepA), making it readily amendable to plant transformation. Rep/RepA encodes a replication initiator protein (Rep) that facilitates rolling-circle replication and circularization of the *BeYDV* genome by creating a single-strand nick within a hairpin structure of the long intergenic region (LIR). Once circularized, the single-stranded *BeYDV* genome can be converted to a double-stranded intermediate and act as a repair template for HR (Baltes et al., 2014).

Previous studies have combined the essential *cis*-acting elements of the *BeYDV*, the LIR and short intergenic region (SIR) into a LIR-SIR-LIR (LSL) orientation within a T-DNA backbone (pLSL T-DNA) with Rep/RepA delivered on a separate construct to generate geminivirus replicons (GVRs) within tobacco leaf cells (Mor et al., 2003; Zhang and Mason, 2006). More recently, co-delivery of a pLSL T-DNA containing both SSNs and repair template with Rep/RepA was shown to coordinate SSN expression with increased repair template copy number in the plant nucleus, and improve the gene targeting efficiency of an integrated reporter in tobacco (Baltes et al., 2014). The reporter system used in this study, referred to as GUPTII, was designed to be used in combination with the efficient SSN, Zif268 and provides a potent tool for assessing HR efficiencies in plant systems (Wright et al., 2005).

In this study, the efficacy of GVRs in concert with SSNs was tested in wild-type and constitutively expressing Rep/RepA mutant genotypes of potato (*Solanum tuberosum* Group Tuberosum L.) by modifying both reporter and endogenous targets. Transformed events supported reduced herbicide susceptibility phenotypes and gene targeting modifications incorporated using a repair template. Regeneration of transformed events under high selection for gene targeting modifications resulted in enhanced levels of gene targeting in regenerated events and further reduced susceptibility to herbicide. This study provides a novel approach to gene targeting in a crop species and demonstrates the utility of geminiviruses for genome editing.

## Materials and Methods

### Vector Construction

pLSL, p35S, and Rep T-DNAs used in GVR replication and the GUPTII assays, and Gateway^®^ (Life Technologies, Carlsbad, CA, USA) entry vectors were obtained from Baltes et al. (2014). The pGUPTII reporter and pGUSNptII control were modified from pDW1364 and pDW1273 (Wright et al., 2005), respectively using *Hind*III and *Sac*I sites for restriction enzyme cloning into the Gateway-compatible binary vector, pMDC32 (p35S; Curtis and Grossniklaus, 2003). The pSSA reporter was cloned using PCR amplification of the GUS coding sequence from pBI101 (Jefferson et al., 1987) and restriction enzyme cloning into pENTR^TM^ (Life Technologies, Carlsbad, CA, USA) using *Bam*HI and *Xho*I sites, and subsequently recombined into p35S. *Avr*II and *Nde*I sites were used to clone the potato *ALS1* S642T target site into the pSSA-S642T reporter.

TALENs were constructed using GoldenGate cloning with NΔ152/C63 N- and C-terminal truncations and TALEN coding sequences separated by a T2A translational skipping sequence (Cermak et al., 2011). CRISPR/Cas vectors were constructed with an *Arabidopsis*-optimized *Streptococcus pyogenes* Cas9 and single-guide RNAs were expressed from an *Arabidopsis thaliana* U6 Pol II promoter (Baltes et al., 2014). TALEN and CRISPR/Cas reagents were recombined into pLSL or p35S Gateway-compatible vectors with or without repair templates.

The left and right homology arms for the *ALS1* repair template, RT1 were cloned from X914-10 genomic DNA and fused to a gBlock^®^ (Integrated DNA Technologies, Coralville, IA, USA) containing a 369 bp *ALS1* target sequence with W563L, S642T and silent mutations using overlapping PCR. *Xba*I and *Bam*HI sites in RT1 were used to clone a T2A:NptII fusion downstream of the *ALS1* target sequence to construct RT2. The pLSLm T-DNA was modified from pLSL using *Bam*HI restriction enzyme digestion and removal of the cauliflower mosaic virus 35S promoter. A list of constructs used in the study is provided in the Supplementary Data Sheet.

### Plant Materials

The tetraploid *S. tuberosum* cultivar “Désirée” (Désirée) and diploid breeding line, MSX914-10 (X914-10) were used in the study. X914-10 is a diploid breeding line from the Michigan State University Potato Breeding and Genetics Program produced from a cross between the doubled-monoploid (DM) *S. tuberosum* Group Phureja line used to construct the potato reference genome (PGSC, 2011) and 84SD22, a heterozygous *S. tuberosum* ×*S. chacoense* hybrid breeding line (Felcher et al., 2012). Désirée is a red-skinned tetraploid potato cultivar with high plant transformation efficiency. Désirée and X914-10 genotypes were used for single-strand annealing (SSA) and GUPTII reporter assays, respectively. A list of genotypes used in the study is provided in the Supplementary Data Sheet.

Tissue culture plants used for *Agrobacterium*-mediated transformation and herbicide spray assays were propagated from shoot tip and axillary bud explants (four per box) and grown in Magenta boxes^®^ (Phytotech, Shawnee Mission, KS, USA) using Murashige and Skoog (MS) medium (Phytotech; product # M519) with 3% sucrose on light racks set to 16-h-light/8-h-night photoperiod at 22°C for 3–4 weeks. Tissue-culture plants in herbicide spray experiments were removed from Magenta^®^ boxes, individually weighed, and replaced into the same Magenta^®^ box. One the same day, the weighed tissue-culture plants were either not sprayed (no spray) or sprayed to saturation (approximately 5 mL) using an aqueous solution containing Imazamox PESTANAL^®^ (7 mg/L; Sigma-Aldrich, St. Louis, MO, USA; product # 34227) and a plastic spray bottle (Sigma-Aldrich, St. Louis, MO, USA; product # BAF116340000). After 4 weeks, tissue-culture plants were removed from Magenta^®^ boxes and individually weighed. Change in fresh weight (Δ fresh weight) was calculated and used as a percentage of the no spray controls for each line.

### *Agrobacterium*-Mediated Transformation

Plant transformations for reporter assays and stable transformations were conducted using previously described methods (Paz and Veilleux, 1999). Stable transformation experiments used 180–200 leaf explants per experiment and reporter assays used four leaf explants per biological replication. Leaf explants were prepared from 3- to 4-weeks-old plants and placed on induction media for 5–7 days prior to inoculation with *Agrobacterium tumefaciens* GV3101. Two days post-inoculation, leaf explants were washed with MS medium containing Cefotaxime (250 mg/L) and Timentin (150 mg/L) antibiotics and placed on regeneration media. Hygromycin (5 mg/L; Sigma-Aldrich, St. Louis, MO, USA; product # 10687) selection was used for primary transformations and reporter assays for T-DNA selection while kanamycin (Sigma-Aldrich, St. Louis, MO, USA; product # K1377) was used for direct selection of gene targeting modifications. Regenerated events were rooted on MS media containing 3% sucrose and the same selection used in regeneration media.

### PCR Detection and Cloning

The Expand^TM^ Long Template PCR system (Roche, Basel, Switzerland; product # 11681834001) was used to detect circularized GVRs and conduct quantitative end-point PCR using NB_415 and NB_416 primers (675 bp). For all other PCR, the Phusion^®^ High-Fidelity DNA Polymerase (New England Biolabs, Ipswich, MA, USA; product # M0531) was used in combination with the following primers: LSL_F and NB_416 were used for pLSL T-DNA (592 bp), GUS_F and GUS_R for GUSNptII (1,835 bp), ALS_mutF2 and ALS_mutR2 for *ALS1* (448 bp), ALS_mutF2 and ALS_R for restriction digestion assays (2,354 bp), ALS_5prime_F and ALS_5prime_R for screening secondary events (2,241 bp), and ALS_3prime_F and ALS_3prime_R for screening regeneration events (1,815 bp). A list of primers and sequences are provided in the Supplementary Data Sheet.

All PCR were run using 100 ng genomic DNA purified from young, fully emerged leaves of transformed/regenerated events or wounded areas of leaf explants using the DNeasy Plant Kit (Qiagen, Venlo, Limburg; product # 69104). Individual plants were used as biological replications in herbicide spray experiments. Leaf explants were sampled by excising wounded areas using a sterile scalpel blade and sampled from three to four explants for a single biological replication. PCR bands were quantified using ImageJ software^1^, normalized to *ALS1* and corrected for size by multiplying the target band intensity by the size ratio of the target band by the *ALS1* band. PCR products were cloned using the Topo^®^ TA cloning kit (Life Technologies, Carlsbad, CA, USA; product # 450071).

### GUS Quantification and Statistics

GUS staining was conducted using X-gluc (5-bromo-4-chloro-3-indoyl-β-D-glucuronic acid) and previously described methods (Butler and Hannapel, 2012). GUS activity quantification was conducted using MUG (4-methylumbelliferyl-β-D-glucuronide) following previously described methods (Jefferson et al., 1987). Protein samples were quantified using Protein Assay Dye Reagent (Bio-Rad, Hercules, CA, USA; product # 5000001) using BSA as a standard (New England Biolabs, Ipswich, MA, USA; product # B9000S). Samples for GUS activity quantification were blanked using time zero samples and read on a Synergy H1 Hybrid plate reader (BioTek, Vinooski, VT, USA). *P*-values were generated using a two-tailed Student’s *t*-test.

## Results

### GVR Delivery and Heterologous Protein Expression

**FIGURE 1:**
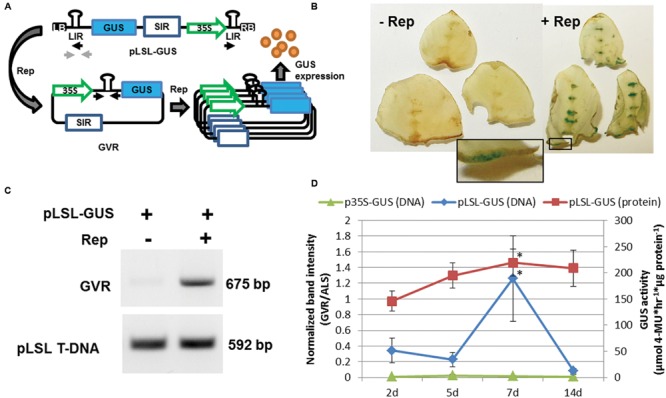
**Delivery of the geminivirus replicon (GVR) to potato leaf explants. (A)** Schematic of pLSL-GUS T-DNA used for *Agrobacterium*-mediated delivery of GVRs to potato leaf tissues. Replicase (Rep) is delivered on a separate p35S T-DNA binary vector (not shown). LB and RB; left and right T-DNA borders, respectively. SIR and LIR; short and long intergenic regions, respectively. 35S; cauliflower mosaic virus promoter. Blue rectangle; GUS coding sequence. Black and light gray arrows; priming sites used for PCR detection of circularized GVRs and pLSL T-DNA, respectively. **(B)** GUS staining of potato leaf explants transformed with pLSL-GUS. Potato leaf explants were transformed with pLSL-GUS in the presence (+Rep) or absence (-Rep) of Rep, and stained for GUS activity 7 days post-inoculation (dpi). Inset is magnification of wounded areas (open black rectangle). Images are from Désirée. **(C)** PCR detection of circularized GVRs in potato leaf explants transformed with pLSL-GUS. Leaf explants transformed with pLSL-GUS in the presence (+) or absence (-) of Rep were sampled for PCR detection of circularized GVRs (675 bp), and the pLSL T-DNA (592 bp) using priming sites from panel **(A)**. Images are from Désirée. **(D)** Time-course of GVRs in potato leaf explants constitutively expressing Rep. Leaf explants prepared from a mutant potato line, D52 (Supplementary Figure S2) were transformed with pLSL-GUS and control p35S-GUS T-DNAs, and sampled after 2, 5, 7, and 14 dpi for quantitative end-point PCR of circularized GVRs (DNA; primary axis) and GUS activity quantification (protein; secondary axis). Error bars represent standard deviations from three biological replications. ^∗^*P* < 0.05; 2 dpi.

A deconstructed mild strain of the *BeYDV* has recently been used to construct an *Agrobacterium* T-DNA capable of delivering GVRs to plant cells (Baltes et al., 2014). This so-called pLSL T-DNA contains two viral *cis*-acting elements required for *BeYDV* replication, the LIR and SIR with a single *trans*-acting element, Rep/RepA delivered on a separate T-DNA (Rep). The L-S-L arrangement of the LIR and SIR elements on the pLSL T-DNA with concomitant expression of Rep/RepA should facilitate replicational release and GVR circularization allowing joining of the two LIR elements of *BeYDV* replicons within plant cell nuclei (Stenger et al., 1991; **Figure 1A**).

To test if GVRs could replicate and express heterologous protein in potato leaf cells, a pLSL T-DNA expressing β-glucuronidase (pLSL-GUS) was used for *Agrobacterium*-mediated transformation of wild-type tetraploid (cv. Désirée) potato leaf explants and whole explants were stained for GUS activity (**Figure 1B**) and sampled for detection of GVR circularization (**Figure 1C**). When concomitantly delivered with Rep, strong GUS staining and GVR circularization were detected in cells of wounded areas (**Figure 1B**; inset and **Figure 1C**; GVR). However, in the absence of Rep, little staining or circularization was observed. Replication and protein expression was further tested in wild-type diploid potato (X914-10) leaf explants. To detect replication levels, quantitative end-point PCR was performed on total DNA isolated after 2, 5 and 7 days post-inoculation (dpi; Supplementary Figure S1). To detect protein expression, a quantitative GUS activity assay was performed. We observed both increased replicon copy numbers and increased GUS expression in samples with Rep co-delivered, relative to samples without Rep. Furthermore, we observed peak replicon levels at ∼5 dpi and peak protein expression ∼7 dpi. These observations suggest Rep/RepA induces efficient heterologous protein expression and replication of GVRs in potato leaf cells.

The requirement of Rep/RepA expression for efficient GVR replication and heterologous protein production led us to question if a constitutively expressing Rep/RepA mutant could be developed in potato. To test this, the constitutively expressing Rep T-DNA was used for stable transformation in X914-10 using hygromycin selection for T-DNA integration. Twenty-eight hygromycin-resistant events were propagated in tissue-culture and used in GVR replication assays to identify an event capable of supporting high GVR replication (Supplementary Figure S2). Among the events evaluated, event D52 displayed both strong GUS staining (data not shown) and high GVR circularization and was chosen for further experimentation. Unlike wild-type X914-10 and Désirée, D52 supported a significant threefold induction of GVR circularization 7 dpi (vs. 5 dpi; *P* < 0.05) and significant heterologous protein production peaking at 7 dpi, similar to wild-type (**Figure 1D** and Supplementary Figure S1; *P* < 0.05) compared to 2 dpi treatments. The difference in GVR circularization between wild-type X914-10 and D52 is likely due to variation in Rep/RepA expression and interactions with the pLSL-GUS T-DNA. In subsequent experiments, we chose to sample wounded areas 7 dpi and focus on X914-10 genotypes in stable gene targeting experiments.

### Design and Activity of SSNs Targeting the Potato *ALS* Gene

Sequence-specific nucleases have been tested in a number of plant species but data in Solanaceous species, such as potato is limited (Li et al., 2013; Brooks et al., 2014; Lor et al., 2014; Sawai et al., 2014; Clasen et al., 2015). Here, we chose to target the potato *ACETOLACTATE SYNTHASE1* (*ALS1*) gene (PGSC0003DMG400034102) for modification. The 3′ end of the *ALS1* sequence contains two codons (W563 and S642; codons TGG and AGC, respectively) that, when changed to W563L and S642T (codons TTG and ACC, respectively), confer reduced susceptibility to ALS-inhibiting herbicides (Sathasivan et al., 1991). To target this region, one TALEN and one CRISPR/Cas reagent were designed to target sequence near each point mutation site [referred to as TALEN(-), CRISPR(-) for W563L and TALEN, CRISPR for S642T] for a total of four nuclease reagents. The further downstream S642T mutation was focused on due to its proximity to the end of the *ALS1* coding sequence and the opportunity to incorporate new sequence downstream of *ALS1*.

**FIGURE 2:**
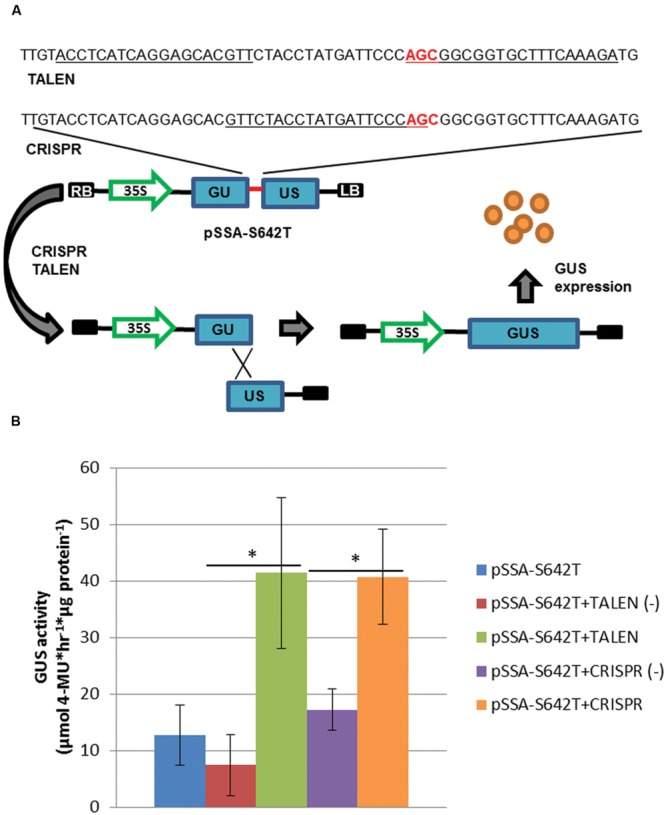
**Sequence-specific nuclease (SSN) activity in potato leaf explants. (A)** Single-strand annealing assay (SSA) incorporating the S642T target site from the potato *ALS1* gene (red line; sequence) delivered on a T-DNA (pSSA-S642T). The SSA reporter cassette was constructed with the GUS coding sequence (GUS) disrupted by a 60 bp S642T target sequence, and a 250 bp direct repeat of the GUS coding sequence (blue rectangles). Binding sites for the p35S-TALEN (TALEN) and -CRISPR/Cas (CRISPR) reagents targeting S642T are underlined with the S642T codon in red. LB and RB; left and right T-DNA borders, respectively. 35S; cauliflower mosaic virus promoter. **(B)** GUS activity quantification of potato leaf explants transformed with pSSA-S642T and SSN reagents. Leaf explants were prepared from X914-10 and transformed with the pSSA-S642T reporter, and TALEN and CRISPR SSN reagents targeting the S642T target site, or negative control p35S-TALEN [TALEN(-)] and -CRISPR/Cas [CRISPR(-)] reagents targeting a heterogeneous potato *ALS1* target site. Error bars represent standard deviations from three biological replications. ^∗^*P* < 0.05; negative control.

To test the activity of TALEN and CRISPR reagents, a SSA reporter was constructed (**Figure 2**; Zhang et al., 2013). The SSA reporter was designed to harbor the S642T target site (pSSA-S642T), where TALEN(-) and CRISPR(-) reagents were used as negative controls. The S642T target site was flanked by a 250 base pair (bp) direct repeat of the GUS coding sequence. Upon formation of a double-strand break within the target site, the SSA direct repeat is used to repair the reporter and reconstitute the GUS coding sequence, allowing GUS expression (**Figure 2A**). To test this system, leaf explants were concomitantly transformed with the pSSA-S642T reporter and SSN reagents (**Figure 2B**). TALEN and CRISPR reagents resulted in significant 5 and 2-fold increases, respectively in reporter activity compared to negative controls (**Figure 2B**; *P* < 0.05). These results suggest the TALEN and CRISPR reagents are active in potato cells and could putatively be used for gene targeting.

The reporter activity using the TALEN reagents suggested TALENs may be capable of inducing targeted NHEJ mutations in transformed events. To investigate this possibility, X914-10 and D52 genotypes were transformed with pLSL-TALEN and p35S-TALEN T-DNAs, respectively and hygromycin-resistant events were screened for targeted NHEJ mutations (not shown). None of the 26 and 42 events generated from p35S-TALEN or pLSL-TALEN contained NHEJ mutations at or above the 6.25% mutation detection limit. The lack of detectable NHEJ mutations in events transformed with TALEN reagents could be due to the formation of somatic mutations that fall below detection limits (Lor et al., 2014).

### GVR-Mediated Gene Targeting

**FIGURE 3:**
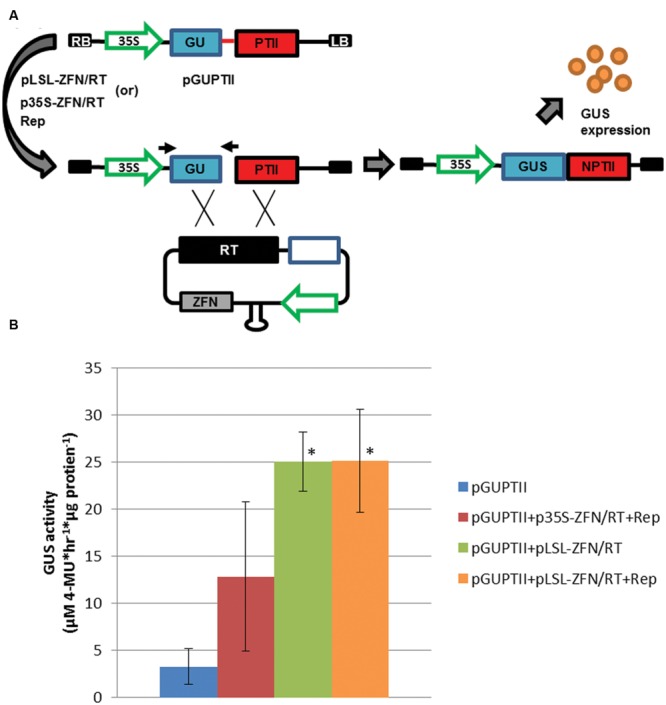
**Gene targeting efficiency in potato leaf explants. (A)** GUPTII reporter assay incorporating the Zif268 target site delivered on a T-DNA (pGUPTII). The GUPTII reporter cassette was constructed with the GUS:NptII translational fusion coding sequence (GUSNptII) disrupted by a 600 bp deletion (blue and red rectangles) and a 60 bp Zif268 target site (red line; Wright et al., 2005). pLSL and p35S T-DNAs incorporating the Zif268 SSN coding sequence (ZFN), and a repair template (RT) incorporating the 600 bp missing sequence and flanking sequence homologous to the GUPTII reporter (black rectangle; Baltes et al., 2014). LB and RB, left and right T-DNA borders, respectively. 35S; cauliflower mosaic virus promoter. Open blue rectangle; short intergenic region (SIR). Gray rectangle; Zif268 coding sequence (ZFN). Black arrows; priming sites used for PCR detection of the repaired pGUPTII reporter (GUSNptII). **(B)** GUS activity quantification of potato leaf explants transformed with pGUPTII and gene targeting reagents. Leaf explants were prepared from Désirée and transformed with the pGUPTII reporter, and p35S and pLSL-ZFN T-DNA gene targeting reagents (p35S-ZFN/RT and pLSL-ZFN/RT) in the presence (+Rep) or absence of Rep/RepA. Rep/RepA was delivered on a 35S T-DNA (Rep). Error bars represent standard deviations from three biological replications. ^∗^*P* < 0.05; pGUPTII.

The demonstration of GVR replication and heterologous protein expression along with SSN activity suggested GVRs were capable of delivering gene targeting reagents to potato cells. In order to test gene targeting efficiencies, a previously established reporter (referred here as pGUPTII) was concomitantly transformed with conventional (p35S) and GVR (pLSL) gene targeting reagents incorporating the Zif268 SSN (ZFN), to potato leaf explants and evaluated for GUPTII reporter repair (GUSNPTII; **Figure 3**). The GUPTII reporter was designed with a 600 bp deletion within the GUS:NptII fusion coding sequence and the Zif268 target sequence in place of the missing sequence (**Figure 3A**). Upon gene targeting, the GUPTII repair template (RT) with the missing 600 bp and flanking homologous sequence is capable of reconstituting the GUS:NptII coding sequence and allowing GUS expression and PCR detection of GUSNptII (Wright et al., 2005).

To determine if gene targeting is enhanced by GVR reagent delivery, pLSL-ZFN/RT and 35S-ZFN/RT reagents were concomitantly delivered with the pGUPTII reporter in the presence of Rep and wounded areas analyzed for GUS activity and GUSNptII detection (**Figure 3** and Supplementary Figure S3). Rep was delivered with both conventional and GVR reagents to normalize Rep/RepA pleiotropic effects (Baltes et al., 2014). GVR delivery of the gene targeting reagents resulted in a significant eightfold increase in reporter activity over the pGUPTII control while no significant increase was observed using the conventional gene targeting reagents (**Figure 3B**; *P* < 0.05). These results were further supported by PCR detection of the repaired GUSNptII detection gene (Supplementary Figures S3A,B). Interestingly, GVR reagents, either with or without Rep, were capable of significant increases in gene targeting (**Figure 3B**; *P* < 0.05). These results may suggest that Rep expression is not required to enhance gene targeting frequencies in potato. However, the sensitivity of the assay combined with the efficiency of transformation in potato leaves may not be sufficient to accurately determine differences between these two treatments. Further, and as expected, we found that GVRs without a functional SSN or repair template fail to mediate efficient gene targeting (Supplementary Figures S3C,D). Collectively, these results indicated that our T-DNA vectors and SSNs were functional, and they were ready to be tested in whole plant assays.

### GVR-Mediated Gene Targeting of the *ALS1* Locus

**FIGURE 4:**
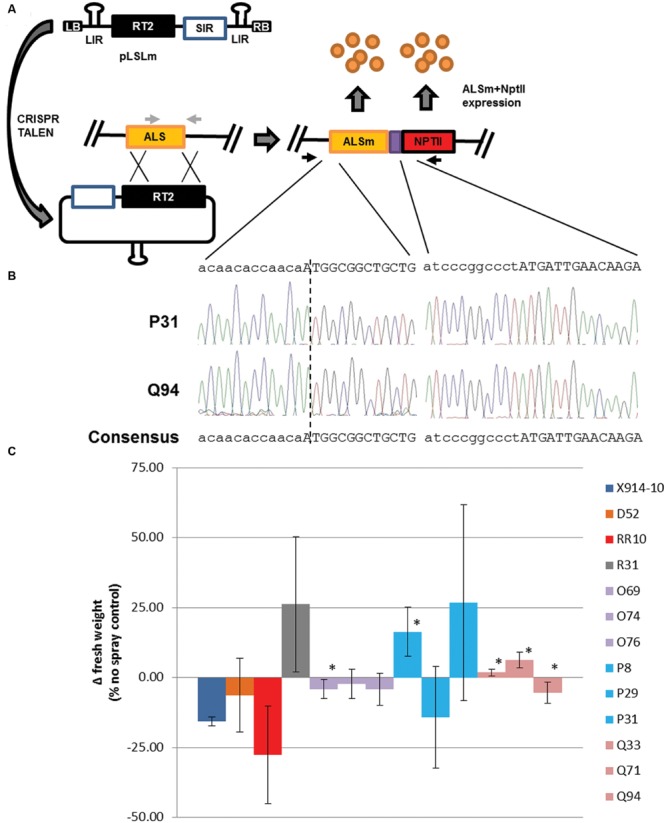
**Geminivirus replicon-mediated gene targeting of the potato *ALS1* gene and herbicide susceptibility in secondary events. (A)** Gene targeting modification of the potato *ALS1* gene (ALS; orange rectangle) using GVRs with (pLSL; **Figure 1A**) or without (pLSLm) SSNs. LB and RB; left and right T-DNA borders, respectively. RT2; *ALS1* repair template. ALSm; modified *ALS1* locus with W563L and S642T mutations and T2A:NptII fusion (purple and red rectangles). SIR and LIR; short and long intergenic regions, respectively. Black arrows; priming sites used for PCR detection (Supplementary Figure S5) and cloning **(B)** of the modified *ALS1* locus. Light gray arrows; priming sites used for PCR detection of the endogenous *ALS1* (Supplementary Figure S2), and gene targeting modification digest assays (Supplementary Figure S4). **(B)** Cloned gene targeting modifications of the *ALS1* gene in secondary events. PCR was used to clone the locus-template junction (left sequences), both W563L and S642T mutations (not shown), and incorporated T2A:NptII (right sequences). Dotted line; locus-template junction. Uppercase sequence: coding sequences for *ALSm* (left) and *NptII* (right). Sequencing traces; P31 (top) and Q94 (bottom). **(C)** Herbicide susceptibility in secondary events. An herbicide spray assay was used to determine herbicide susceptibility in wild-type (X914-10), primary (D, R lines), and secondary events (RR, O, P, Q lines). Primary events were generated by transforming X914-10 with Rep (D52) or the *ALS1* transgene (R31), and applying hygromycin selection. Secondary events were generated by transforming D52 with p35S-TALEN/RT2 (RR10), pLSLm+CRISPR (O69, O74, O76), pLSLm+TALEN (P8, P29, P31), or pLSL-TALEN/RT2 (Q33, Q71, Q94) and applying 50 mg/L kanamycin (Kan50) selection. Change in fresh weight (Δ fresh weight) was calculated as a percentage of the no spray controls for each line. Error bars represent standard deviations from three biological replications. ^∗^*P* < 0.05; X914-10.

The ability of GVRs to modify the GUPTII reporter in potato and tobacco leaf cells suggests GVRs could also modify an endogenous locus (Baltes et al., 2014). The *ALS1* locus provides an ideal target for gene targeting given the ubiquitous nature of *ALS* expression and the availability of ALS-specific point mutations conferring reduced herbicide susceptibility in a broad range of plant species (Endo and Toki, 2013). SSNs developed to target the S642T point mutation site in the potato *ALS1* gene were cloned into both p35S and pLSL T-DNAs along with an *ALS1* repair template. The *ALS1* repair template, referred here as RT1 was first constructed to contain both W563L and S642T point mutations and silent mutations within TALEN and sgRNA binding sites to prevent SSN off-targeting of the repair template. Left and right homology arms were designed to cover the *ALS1* coding sequence stopping at a non-functional start codon [1.6 kilobases (kb)] and 1 kb downstream of the *ALS1* coding sequence, respectively. A *Bam*HI restriction enzyme site at the end of the *ALS1* coding sequence was used for both screening for gene targeting modifications in transformed events (Supplementary Figure S4) and constructing a second *ALS1* repair template, RT2 which includes a T2A:NptII translational fusion (**Figure 4**). RT2 was also used to construct an *ALS1* transgene which incorporates all the modifications in RT1 and -2 but contains a functional *ALS1* coding sequence (*ALSm*) that is driven by a 2.5 kb promoter region upstream of the endogenous *ALS1* coding sequence.

Stable plant transformations were conducted using the constitutively expressing Rep mutant, D52 to test the efficacy of the gene targeting reagents. D52 was chosen for gene targeting transformations to simplify reagent delivery, normalize the pleiotropic effects of Rep, and potentially improve transformation efficiency (Baltes et al., 2014). Preliminary transformations were conducted with TALEN and RT1 reagents in both the wild-type X914-10 and D52 backgrounds without direct selection for gene targeting modifications, relying on the efficiency of the reagents (Supplementary Figure S4). Primary transformations were conducted using X914-10 with p35S-TALEN/RT1 reagents and secondary transformations using D52 with pLSL-TALEN/RT1 reagents. Hygromycin-resistant events were screened for both gene targeting modifications and NHEJ targeted mutations using restriction digestion and T7 endonuclease I (T7EI) assays, respectively (Huang et al., 2012). None of the 72 primary and 78 secondary events contained any detectable gene targeting modifications or NHEJ mutations. These results reflect the previous evaluation of the TALEN reagents for producing NHEJ mutations in stable events (data not shown) and support findings in tobacco and potato that GVRs do not improve NHEJ frequencies (Baltes et al., 2014; Butler et al., 2015). Furthermore, the lack of detectable gene targeting modifications within transformed events suggested direct selection for gene targeting and more sensitive detection methods were needed in the event somatic modifications were being formed (Feng et al., 2014).

### Recovery of *ALS1* Modified Events Using Direct Selection

In order to improve gene targeting efficiency in transformed events, new gene targeting reagents were developed incorporating RT2 to allow direct selection for gene targeting modifications and to deliver RT2 independently on GVRs using the D52 background (**Figure 4A**). The T2A:NptII translational fusion used in RT2 allows for independent function of both ALSm and NptII proteins in modified cells, supporting resistance to both ALS-inhibiting herbicides and kanamycin, respectively. In addition to the GVR pLSL-TALEN/RT2 reagent, a p35S-TALEN/RT2 reagent was also constructed to compare conventional T-DNA and GVR delivery in secondary transformations. Furthermore, a modified pLSL (pLSLm) T-DNA was constructed that incorporates RT2 but does not include a 35S promoter or SSN reagents. Our reasoning was that by delivering RT2 on a GVR (pLSLm) and SSNs on a separate 35S T-DNAs (TALEN and CRISPR), gene targeting efficiency could be improved by altering the coordination of SSN expression and repair template availability. This approach was supported by findings in tobacco where gene targeting efficiencies were significantly enhanced by delivering a repair template on a GVR compared to SSNs (Baltes et al., 2014).

The new gene targeting reagents were tested in D52 secondary plant transformations using kanamycin selection (50 mg/L; Kan50; **Table 1**). Transformations were carried out using pLSL and p35S reagents by transforming pLSL-TALEN/RT2 and p35S-TALEN/RT2 in two replicate experiments, resulting in 12 Kan50 resistant events (Q lines) and 4 Kan50 resistant events (RR lines). Transformations were carried out using the pLSLm T-DNA by transforming pLSLm and either TALEN (13 Kan50 resistant events; P lines) or CRISPR (8 Kan50 resistant events; O lines) in two replicate experiments. Kan50 resistant lines were screened using PCR detection of gene targeting modifications in leaf tissues (Supplementary Figure S5). Bands generated from Kan50 resistant lines could not be clearly seen. To improve detection, band quantification was used to identify lines with band intensities at least twofold higher than *ALS1* internal controls. Using this criterion, 41.7 and 12.5% of the Q and O lines, respectively were determined as being positive for gene targeting modifications while none of the RR or P lines were above this threshold. To validate the bands generated in the screen, two representative lines, P31 and Q94 were chosen for gene targeting modification cloning (**Figure 4B**). PCR products from both representative lines were cloned and sequenced. In both cases, new sequence incorporated by the repair template was identified and linked to the template-locus junction. These results confirm the validity of bands used for evaluating gene targeting events and the likely presence of somatic gene targeting modifications within events.

**Table 1:** Summary of gene targeting screens of secondary and regenerated events.

T-DNA	Total Kan50 resistant events	Kan50 resistant lines	# PCR positive events (secondary)	% PCR positive events (secondary)	Total Kan100 resistant events	Kan100 resistant lines	# PCR positive events (regeneration)	% PCR positive events (regeneration)
p35S-TALEN/RT2	4	RR	0	0%	0	(none)	0	0%
pLSLm+CRISPR	8	O	1	12.5%	31	DD	10	32.2%
pLSLm+TALEN	13	P	0	0%	27	EE	9	33.3%
pLSL-TALEN/RT2	12	Q	5	41.7%	29	FF	10	34.5%

*Secondary events transformed with conventional (p35S) and GVR (pLSL and pLSLm) T-DNA (T-DNA) carrying TALEN and CRISPR reagents (**Figure 2A**) and the *ALS1* repair template containing NptII (RT2) capable of rooting in kanamycin 50mg/L media were selected as resistant events (Total Kan50 resistant events). A PCR assay was used to identify secondary events with gene targeting modifications (# PCR positive events (secondary)) and is shown as a percentage of total Kan50 resistant events (% PCR positive events (secondary)). Regenerated secondary events capable of rooting in kanamycin 100 mg/L media were chosen as resistant events (Total Kan100 resistant events). A PCR assay was used to identify regeneration events with gene targeting modifications (# PCR positive events (regeneration; Supplementary Figure S6)) and is shown as a percentage of total Kan100 resistant events (% PCR positive events (secondary; Supplementary Figure S5)). Secondary events were generated using the D52 genotype and DD, EE, and FF regenerated events were generated using the O74, P31, and Q94 genotypes, respectively (Supplementary Data Sheet).*

The confirmation of gene targeting modifications, including W563L and S642T point mutations, and kanamycin resistance of secondary events suggested the events may also display reduced susceptibility to ALS-inhibiting herbicides. To test this, an imidizolinone herbicide was used to spray tissue-culture plants and changes in fresh weight as a percentage of non-sprayed controls were compared to X914-10 wild-type and *ALSm* transgenic line, R31, 4 weeks post-spraying (**Figure 4C**). R31 maintained approximately 25% positive growth, while both D52 and RR10 (p35S-TALEN/RT2) showed no significant difference from X914-10, ranging from 16 to 27% negative growth. O lines were also similar to X914-10 but showed significant growth in one line (O69; *P* < 0.05). P lines showed growth variability across lines, ranging from 14% negative to 27% positive growth with one line showing significant positive growth (P8; *P* < 0.05). Q lines had less variability than P lines with all three evaluated lines showing significantly improved growth from X914-10 and two lines having positive growth (Q33 and Q71; *P* < 0.05). Overall, five of the nine evaluated events (56%) displayed reduced susceptibility to imidizolinone herbicide and 18% of the kanamycin resistant events had detectable gene targeting modifications. These results support the use of GVRs for promoting gene targeting modifications of an endogenous locus in secondary events and gene targeting efficacy of TALEN and CRISR/Cas reagents delivered by GVRs.

### Enhancement of Gene Targeting Modification Using Regeneration

**FIGURE 5:**
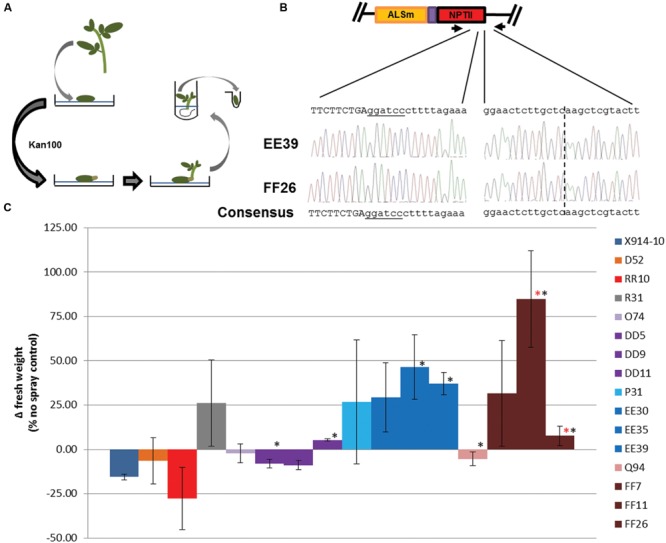
**Regeneration of events on high kanamycin selection and herbicide susceptibility in regenerated events. (A)** Regeneration of secondary events on high kanamycin selection. Individual events were used for regeneration on kanamycin 100 mg/L (Kan100) selection media. Regenerated events capable of rooting in Kan100 where used for cloning the gene targeting modified *ALS1* locus, **(B)** and in herbicide susceptibility experiments **(C)**. ALSm; modified *ALS1* locus with W563L and S642T mutations and T2A:NptII fusion (purple and red rectangles). **(B)** Cloned gene targeting modifications of the *ALS1* gene in regenerated events. Primers specific to the gene targeting modified *ALS1* locus (black arrows) were used for PCR (Supplementary Figure S6), and to clone the template-locus junction (right sequences) and incorporated *NptII* (left sequences). Dotted line; template-locus junction. Uppercase sequence; *NptII* coding sequence. Underlined sequence; *Bam*H1 site. Sequencing traces; EE39 (top) and FF26 (bottom; **C)** Herbicide susceptibility in regenerated events. An herbicide spray assay was used to determine herbicide susceptibility in wild-type (X914-10), secondary (D52, RR10, R31, O74, P31, Q94), and regenerated events (DD, EE, FF lines). Wild-type, primary and secondary events data comes from **Figure 4**. Regenerated events originated from O74 (DD5, DD9, DD11), P31 (EE30, EE35, EE39), and Q94 (FF7, FF11, FF26) secondary events. Error bars represent standard deviations from three biological replications. ^∗^*P* < 0.05; X914-10. ^∗^*P* < 0.05; Q94.

Previous studies have shown production of somatic mutations and chimerism is common among plant events transformed with SSN reagents (Gao et al., 2010; Feng et al., 2014; Wang et al., 2014). The difficulty to detect gene targeting modifications in secondary events suggested this may also be the case in potato (Supplementary Figure S5). A recent study in a related species, tomato (*Solanum lycopersicum*) used multiple rounds of regeneration and SSN induced expression to enhance levels of SSN-mediated modifications in primary events (Lor et al., 2014). To test this approach, three events derived from each p35S, pLSL, and pLSLm reagent were used for regeneration on high kanamyacin selection (100 mg/L; Kan100) in two replicate experiments (**Figure 5A**). Although no Kan100 resistant lines were recovered from X914-10, D52, or p35S-TALEN/RT2-derived events, a similar number of lines were recovered from single events derived from pLSLm+CRISPR (DD lines), pLSLm+TALEN (EE lines) and pLSL-TALEN/RT2 (FF lines) reagents (**Table 1**). Regenerated events were screened for gene targeting modifications using PCR as previously described (Supplementary Figure S6). Unlike secondary events, clear gene targeting modification bands were observed in regeneration events at similar frequencies across reagents. To confirm the bands represent gene targeting modifications, two representative regeneration events, EE39 and FF26 were used for cloning and sequencing the modified locus (**Figure 5B**). Both representative events were confirmed as containing gene targeting modifications which included NptII template-specific sequence linked to the template-locus junction. These results suggest the regeneration of secondary events under high selection for gene targeting modifications was capable of enhancing the level of gene targeting modifications in regenerated lines.

To investigate the phenotypic effects enhanced by gene modifications in regenerated lines, three regenerated lines per reagent were subjected to the herbicide spray assay previously described and compared to X914-10, R31, and progenitor secondary events (**Figure 5C**). In general, regenerated lines showed improvements in reduced herbicide susceptibility compared to progenitor lines. DD lines showed similar growth to its progenitor and other O lines with significant growth in two events (DD5 and DD11; *P* < 0.05). EE lines showed less growth variability compared to its progenitor and other P lines, and displayed positive growth similar to R31, with significant positive growth in two events (EE35 and EE39; *P* < 0.05). FF lines showed the most dramatic improvements in reduced herbicide susceptibility which ranged from 8 to 85% positive growth, with events FF11 and FF26 displaying significant growth improvements over its progenitor (*P* < 0.05). The sustained variation in both gene targeting modifications and reduced herbicide susceptibility phenotypes in secondary and regenerated events reflect the putative somatic nature of these modifications and can be explained by the multicellular origin of shoot organogenesis (Poethig, 1989; Faize et al., 2010; Zhu et al., 2010). These results and previous studies support the use of regeneration to enhance SSN-mediated modification in chimeric events and modified tissues and provide a novel approach to gene targeting in plant species (Marton et al., 2010; Chen, 2011; Lor et al., 2014; Ali et al., 2015).

## Discussion

The utility of plant viruses for delivering genome editing reagents is just being realized in studies using *TRV* and now with geminiviruses (Marton et al., 2010; Baltes et al., 2014; Ali et al., 2015; Čermák et al., 2015). Geminiviruses not only provide strong heterologous protein expression but because of their DNA genomes, can serve as potent repair templates for gene targeting. Furthermore, the broad host range of geniminiviruses, such as the bean yellow dwarf virus allows a number of different model and crop species to be modified using the same essential viral elements. Together with the rapid development SSN technology positions geminiviruses to be become powerful tools for genome editing.

Geminiviruses have historically been used for producing high levels of heterologous protein in plant tissues (Mor et al., 2003; Zhang and Mason, 2006; Chen et al., 2011). It would then seem intuitive that geminiviruses could be harnessed to express SSNs and improve rates of NHEJ in regenerating plant tissues (Baltes et al., 2014). However, no improvements in NHEJ were observed in transformed tissues or in stable events (Supplementary Figure S4). These results suggest that the optimal SSN expression can be achieved with conventional T-DNA or plasmid vectors, and a further increase in SSN expression is not beneficial (Baltes et al., 2014; Butler et al., 2015).

Efficient GVRs is dependent on the expression of the viral *trans*-acting element, Rep/RepA. Rep/RepA has been shown to have pleiotropic effects in both monocot and dicot species which promotes cell-cycling progression of non-dividing cells to enter the S-phase (Ascencio-Ibáñez et al., 2008). This transition provides the virus with the host factors necessary for DNA replication, but also improves rates of regeneration in plant transformation experiments (Gordon-Kamm et al., 2002). The development of a constitutively expressing Rep/RepA expressing potato line made use of both of these benefits of Rep/RepA expression and simplified reagent delivery (Supplementary Figure S2). However, the ability of the geminivirus to facilitate increases in gene targeting frequencies independent of Rep/RepA in potato leaf cells suggests that Rep/RepA may not be required for efficient gene targeting in potato and may only require *cis*-acting elements present on the geminivirus as demonstrated in tobacco (Supplementary Figure S3; Gover et al., 2014). Nevertheless, improved geminivirus systems have been developed which combine Rep/RepA with the geminivirus and further simplify delivery (Baltes et al., 2014; Čermák et al., 2015).

Production of somatic modifications in lines transformed with SSNs has been previously reported in other plants species and using geminivirus delivery (Feng et al., 2014; Richter et al., 2014; Čermák et al., 2015). Putatively, more efficient reagents will be capable of inducing stable germline mutations in primary events (Brooks et al., 2014). However, the drastic differences between frequencies of NHEJ and HR in plant cells makes development of germline HR modified lines more difficult in primary events (Wright et al., 2005). This is even further complicated in vegetatively propagated species, like potato that cannot be taken through the germline without changing cultivar characteristics (Douches et al., 1996).

Selectable makers, such as NptII were used in this study and other studies to facilitate generation of gene targeting events (Baltes et al., 2014; Čermák et al., 2015). This is problematic for development of so-called “clean” gene targeting events which do not carry selection markers and are useful for commercialization. Reliance on selection markers and difficulties with HR may be overcome by utilizing so-called in planta genome editing where HR is allowed to occur during the life of the plant and modifications can be fixed within cell lines (Fauser et al., 2012). This novel approach proved effective to improve HR in potato by putting events through a subsequent round of regeneration, applying selection, and regenerating modified cells (**Figure 5**). However, the use of NptII prevents the resulting regenerated events from being “clean.” This issue could be addressed by using an NptII-free repair template and relying on resistance to ALS-inhibiting herbicides for regeneration. This approach may also provide an opportunity to use *ALS1* as a “trait-landing” site for including other new sequence such as *cis-*genes.

## Author Contributions

NMB and NJB conceived the work plan for the study, wrote the manuscript, and contributed materials and reagents. NMB conducted the experiments and data analysis. NMB, NJB, DV, and DD reviewed, edited and approved the manuscript.

## Conflict of Interest Statement

The authors declare that the research was conducted in the absence of any commercial or financial relationships that could be construed as a potential conflict of interest.

## Footnotes

1 http://imagej.nih.gov/ij/
