# Supplementary material for: Geminivirus-Mediated Genome Editing in Potato (Solanum tuberosum L.) Using Sequence-Specific Nucleases
Source: Front Plant Sci. 2016 Jul 21;7:1045. doi: 10.3389/fpls.2016.01045 (PMC4955380; doi:10.3389/fpls.2016.01045)
Supplement: Supplementary file 1 [file Data_Sheet_1.PDF]

Supplementary material

Geminivirus-mediated genome editing in potato

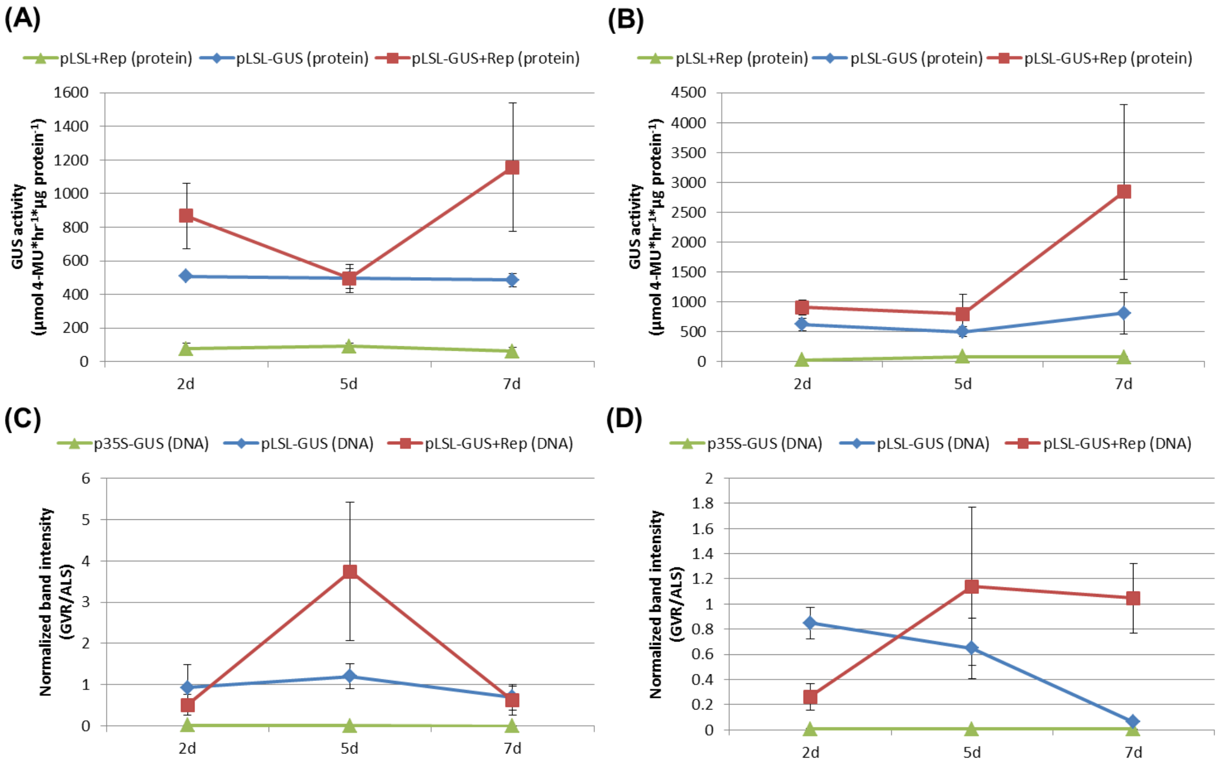

Figure S1 Time-course of GVR replication (DNA) and GVR-mediated protein expression (protein) in wild-type potato leaf explants. Leaf explants prepared from wild-type X914-10 (**A**,**C**) and Désirée (**B**, **D**) were transformed with pLSL-GUS and control T-DNAs (pLSL and p35S-GUS) in the presence (+Rep) or absence of Rep and sampled after 2, 5 and 7 dpi. Leaf explant tissues were sampled for GUS activity quantification (**A**-**B**) and quantitative end-point PCR detection of circularized GVRs (**C**-**D**) (Figure 1). Error bars represent standard deviations from three biological replications.

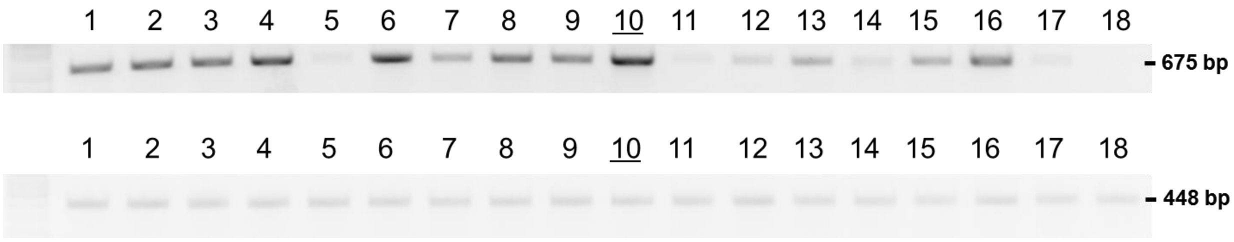

Figure S2 GVR screen of primary potato events transformed with Rep. Leaf explants from hygromycin-resistant primary events transformed with Rep (#1-17) and X914-10 wild-type (#18) were transformed with pLSL-GUS, and leaf explant tissues were collected for PCR detection of circularized GVRs (675 bp; top image) (Figure 1) and *ALS1* (448 bp; bottom image) as an internal control. Primary event, D52 (#10; underlined) was chosen for use in the study.

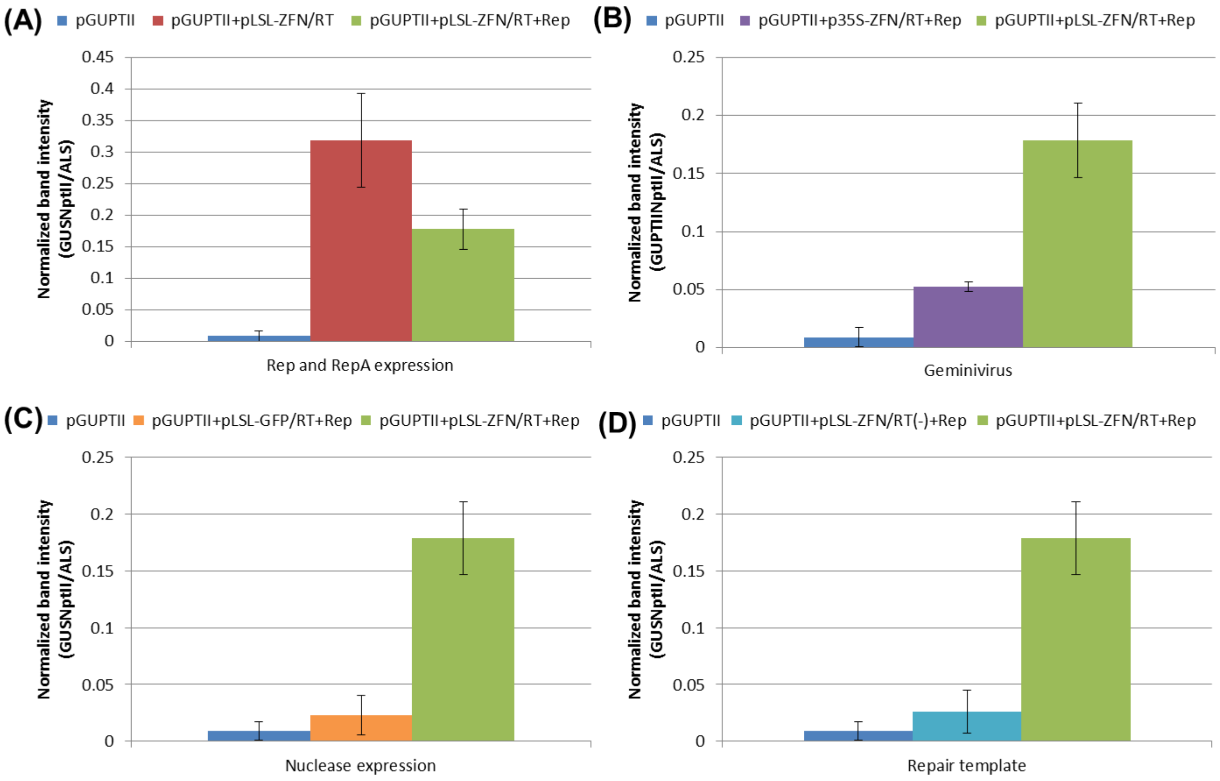

Figure S3 PCR detection of pGUPTII reporter repair in potato leaf explants transformed with gene targeting reagents. Leaf explants prepared from wild-type potato plants were transformed with the pGUPTII reporter and pGUPTII gene targeting reagents (pLSL-ZFN/RT (**A**) and p35S-ZFN/RT (**B**)) in the presence (+Rep) or absence of Rep (Figure 3). Control experiments were conducted using pLSL-GFP/RT (**C**) and pLSL-ZFN/RT(-) (**D**) T-DNAs where the Zif268 coding sequence was replaced with green fluorescent protein (GFP) and the pGUPTII repair template (RT) was replaced with a heterologous tobacco *ALS* RT (RT-), respectively (Baltes *et al*., 2014). Leaf explant tissues were sampled for PCR detection using priming sites shown in Figure 3 (black arrows). Error bars represent standard deviations from three biological replications.

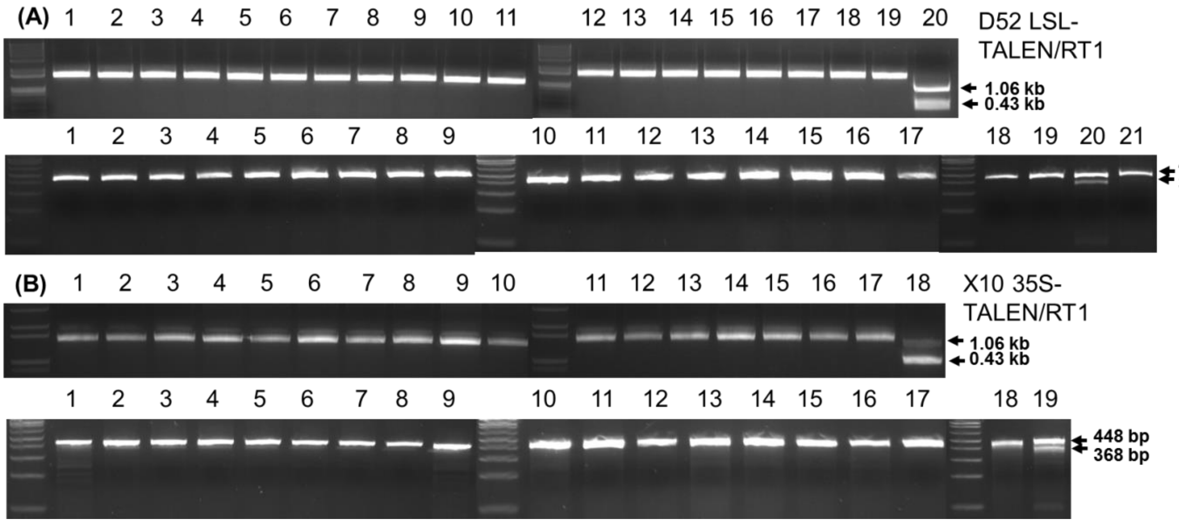

Figure S4 Screen for gene targeting modifications and nonhomologous end-joining (NHEJ) mutations in *ALS1* of primary and secondary events without using direct selection for gene targeting. The *ALS1* repair template (RT1) used for transformation was constructed without T2A:NptII (not shown), and transformations were conducted using hygromycin for T-DNA selection. Secondary events (lanes 1-19) were transformed with D52 using pLSL-TALEN/RT1 T-DNA (**A**), and primary events (lanes 1-17) were transformed with X914-10 using p35S-TALEN/RT1 T-DNA (**B**) and screened for gene targeting modifications (upper gels) or NHEJ mutations (lower gels) in the *ALS1* gene. Gene targeting screening was conducted using a digest assay using priming sites from Figure 4A (light gray arrows) and *Bam*HI restriction enzyme digestion of the PCR amplicon purified using the QIAquick PCR purification kit (Qiagen, Lenlo, Limburg; product # 28104) (Figure 5B). Wild-type amplicons will remain undigested (1.49 kb) while gene targeting modified amplicons will form 1.06 kb and 0.43 kb digest bands (lanes 20 (**A**) and 18 (**B**); black arrows). NHEJ screening was conducted using a T7EI assay using priming sites from Figure 4a and T7 endonuclease I (NEB, Ipwich, MA; product # M0302) digestion (Huang *et al.,* 2012). Wild-type amplicons (448 bp) will remain undigested (lanes 21 (**A**) and 18 (**B**)) while amplicons with NHEJ mutations will form a 368 bp band (lanes 20 (**A**) and 19 (**B**)).

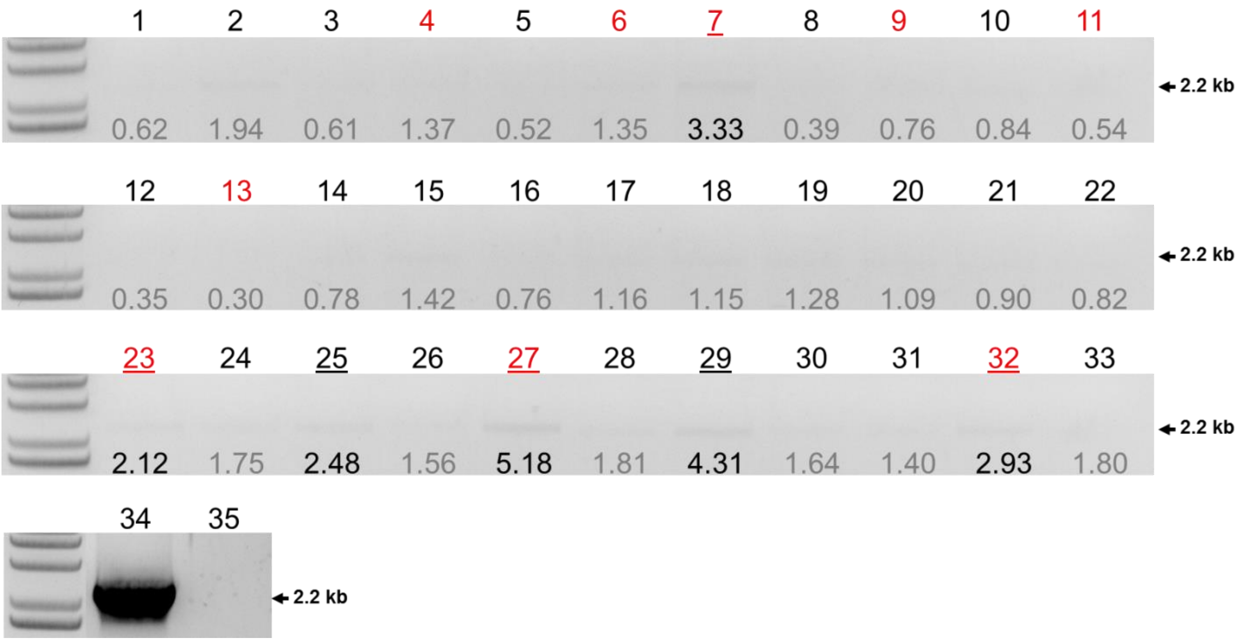

Figure S5 Screen for gene targeting modifications in *ALS1* of secondary events using direct selection for gene targeting. Secondary transformations of D52 were conducted using pLSLm+CRISPR (lanes 1-8), pLSLm+TALEN (lanes 9-21) and pLSL-TALEN/RT (lanes 22-33) with direct selection for gene targeting using kanamycin 50 mg/L. PCR screening was conducted using priming sites specific to the modified *ALS1* locus (Figure 4A; black arrows) with an expected amplicon size of 2.2 kb (lane 34; positive control). PCR products were run on 1.0% agarose gels and band intensities were quantified and normalized to *ALS1* for each secondary event (values). Secondary events were considered positive if they had band intensities equal or more than two-fold *ALS1* (black values, underlined) and nine were chosen for further analysis (red; Figures 4 and 5). D52 was used as a negative control (lane 35).

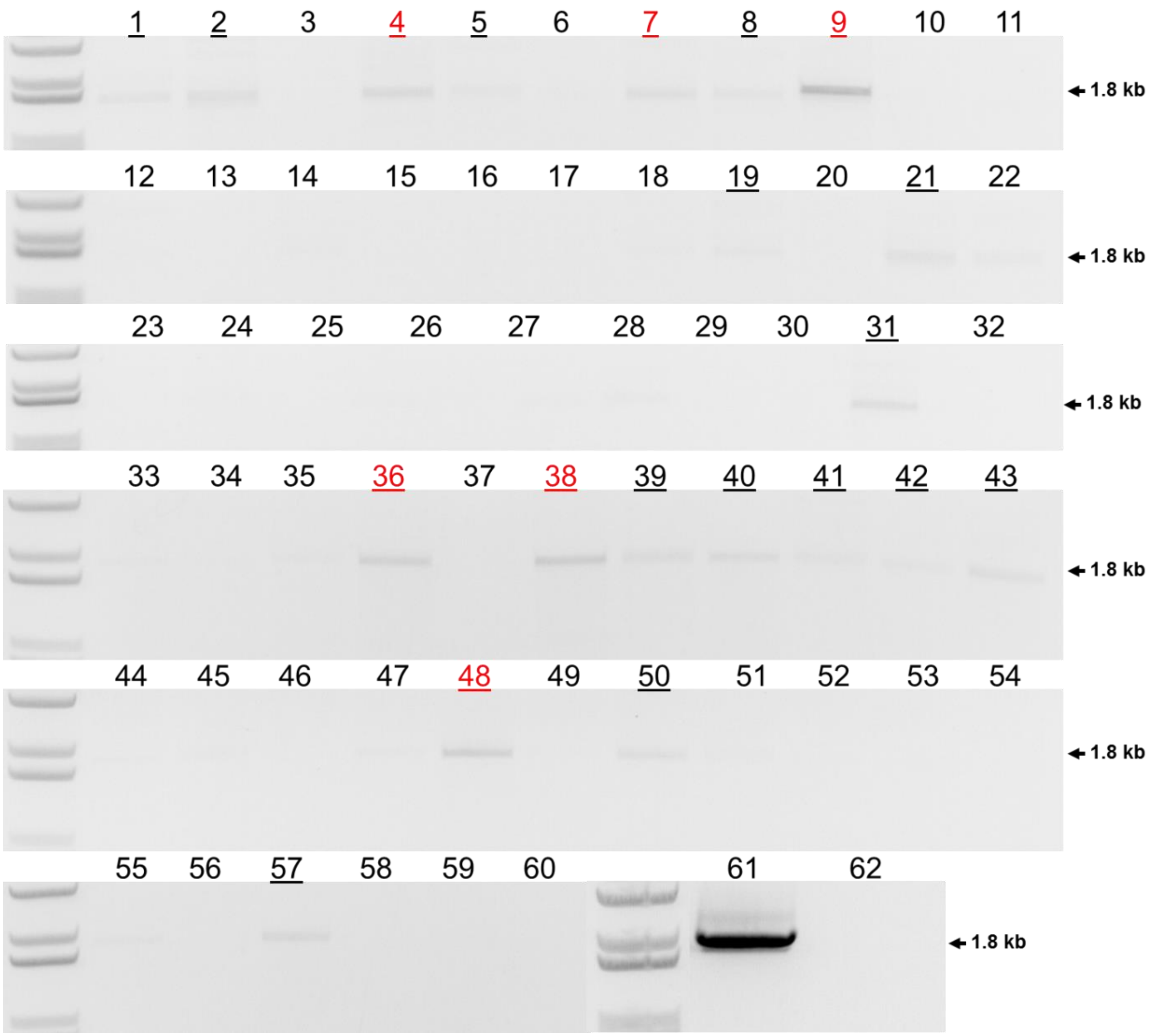

Figure S6 Screen for gene targeting modifications in *ALS1* of regenerated events using high direct selection for gene targeting. Regeneration was conducted using O74 (lanes 1-31), P31 (not shown), and Q94 (lanes 32-60) secondary events with high direct selection for gene targeting using kanamycin 100 mg/L (Figure 5). PCR screening was conducted using priming sites specific to the modified *ALS1* locus (Figure 5B; black arrows) with an expected amplicon size of 1.8 kb (lane 61; positive control). Regenerated events were considered positive if they had visible bands (underlined) and nine were chosen for further analysis (red; Figures 5). D52 was used as a negative control (lane 62).
